# Supplementary material for: Cell-type-specific immune programs orchestrate spatial defense in the Arabidopsis leaf epidermis
Source: Nat Commun. 2026 Mar 21;17:4296. doi: 10.1038/s41467-026-70843-z (PMC13172538; doi:10.1038/s41467-026-70843-z)
Supplement: Supplementary file 1 — Supplementary Information [file 41467_2026_70843_MOESM1_ESM.pdf]

Cell-type-specific immune programs orchestrate spatial defense in the Arabidopsis leaf epidermis

Jingpu Song, Mahsa Modareszadeh, Dinithi Kumarapeli, Wilson Andres Acosta, Yuhai Cui & Yangdou Wei

Supplementary Fig. 1

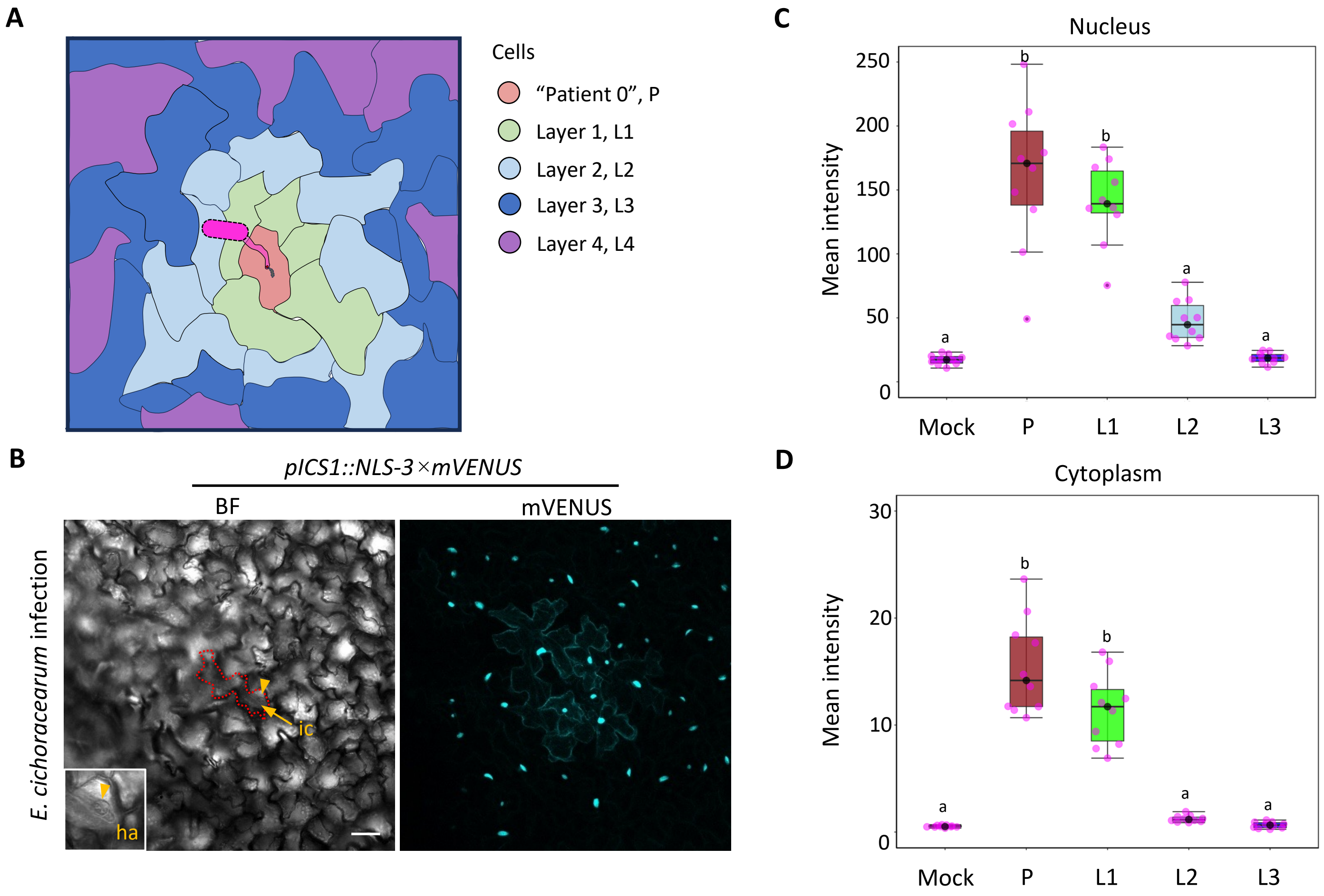

Supplementary Fig. 1 Differential expression of *pICS1::NLS-3×mVENUS* in Arabidopsis leaves in response to adapted powdery mildew infection.

A, Schematic representation of the infection site following single-infection. The initially infected cell, referred to as "Patient 0" (P), is surrounded by successive layers of epidermal cells. Cells in direct contact with P form the first layer (L1), whereas cells adjacent to L1, but not directly bordering P, constitute the second layer (L2), and so forth. **B**, Representative confocal micrographs showing the expression pattern of *pICS1::NLS-3×mVENUS* in Arabidopsis leaf tissues surrounding *Erysiphe cichoracearum* (*E. cichoracearum*)-penetrated sites at 26 hours post inoculation (hpi). Fluorescent mVENUS signals are displayed as maximal intensity projections of Z-stacks. At least 10 single-infection sites were examined. mVENUS signals are shown in cyan. *E. cichoracearum*-penetrated cell is outlined with red dash lines. ic, infected cell; ha, haustorium. Scale bars, 50 µm. Source data are provided as a Source Data file. **C**, Quantitative analysis of nuclear mVENUS signal intensities at *E. cichoracearum*-penetrated sites. The penetrated cell is designated as "Patient 0" (P). The average mean intensity of Lx nuclei was calculated as: sum(mean intensity of individual L1 nucleus)/number of Lx cells, where Lx= L1, L2, and L3. **D**, Quantitative analysis of cytoplasmic mVENUS signal intensities at *E. cichoracearum*-penetrated sites. The average mean intensity of Lx cytoplasm was calculated as: sum(mean intensity of individual L1 cytoplasm)/number of Lx cells, where Lx= L1, L2, and L3. **C**-**D**, All boxplots show the median (center line, n=10), second to third (25% to 75%) quartiles (box), minimum and maximum values (whiskers) of measurement. The small letters a to b denote statistically significant differences between means with one-way ANOVA, followed by the post hoc Tukey multiple comparison tests ($p < 0.05$). All p values are shown in Source data. Source data are provided as a Source Data file.

Supplementary Fig. 2

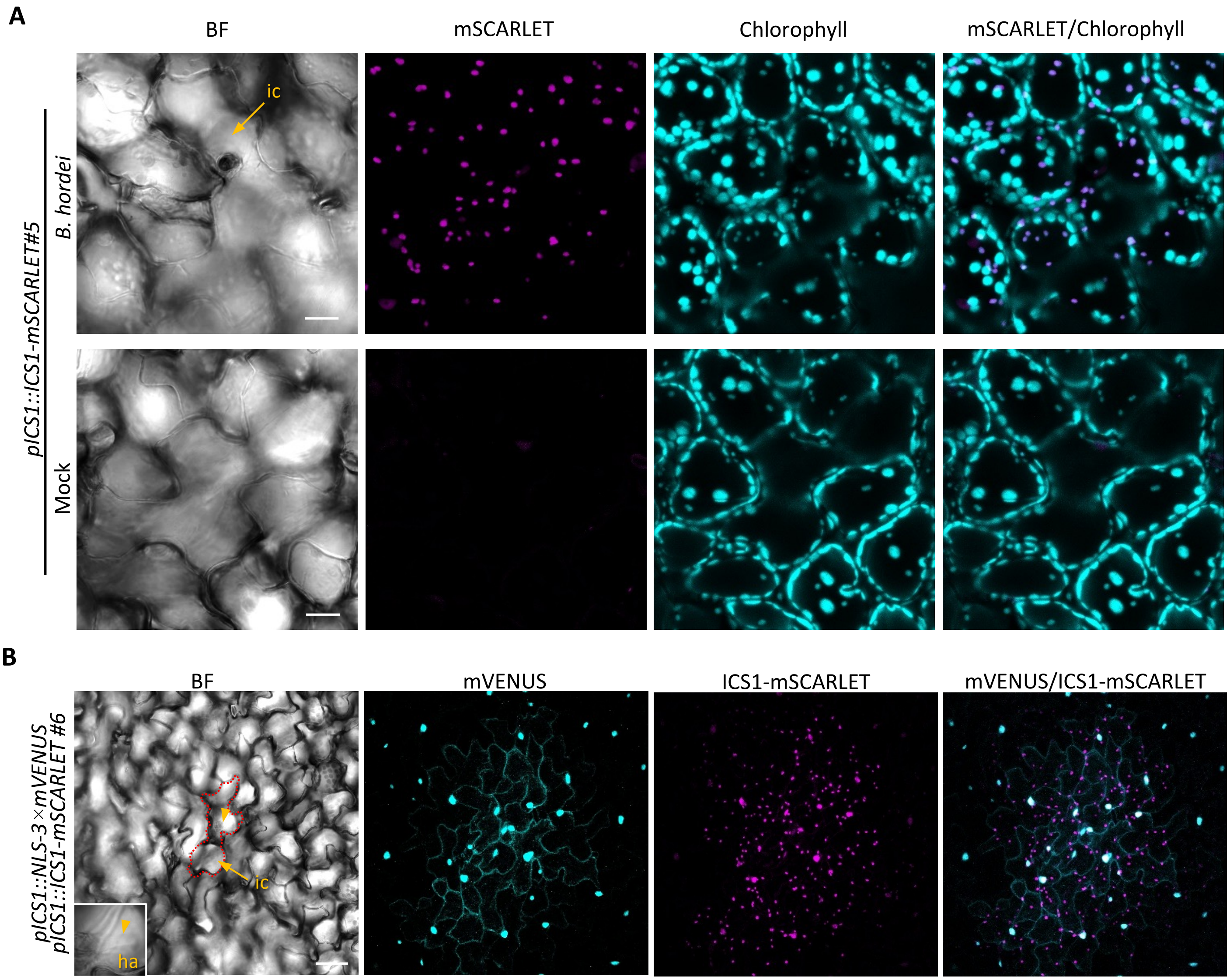

Supplementary Fig. 2 Subcellular localization and induction pattern of ICS1-mSCARLET in response to *Blumeria hordei* infection.

A, Representative confocal images showing the expression pattern of ICS1-mSCARLET in *B. hordei*-infected (24 hpi) and non-infected leaves of *pICS1::ICS1-mSCARLET* Arabidopsis plants. Experiments were repeated three times, with at least ten single-infection sites examined. Fluorescent channels are presented as maximal intensity projections of Z-stacks. mSCARLET signals (ICS1 expression) are shown in magenta, and chlorophyll A autofluorescence is shown in cyan. ic, infected cell; Scale bars, 20 µm. **B**, Representative confocal images showing the expression pattern of *pICS1::ICS1-mSCARLET* in an independent transgenic line (pICS1::ICS1-mSCARLET#6) at 24 hpi following *B. hordei* penetration. Experiments were repeated three times, with at least ten single-infection sites examined. Fluorescent images are presented as maximal intensity projections of Z-stacks. mVENUS signals appear in in cyan, and mSCARLET signals in magenta. Infected cells are outlined with red dash lines, and haustoria indicated by arrowhead is shown in magnified inset for clarity. ic, infected cell; ha, haustorium. Scale bars, 50 µm.

Supplementary Fig. 3

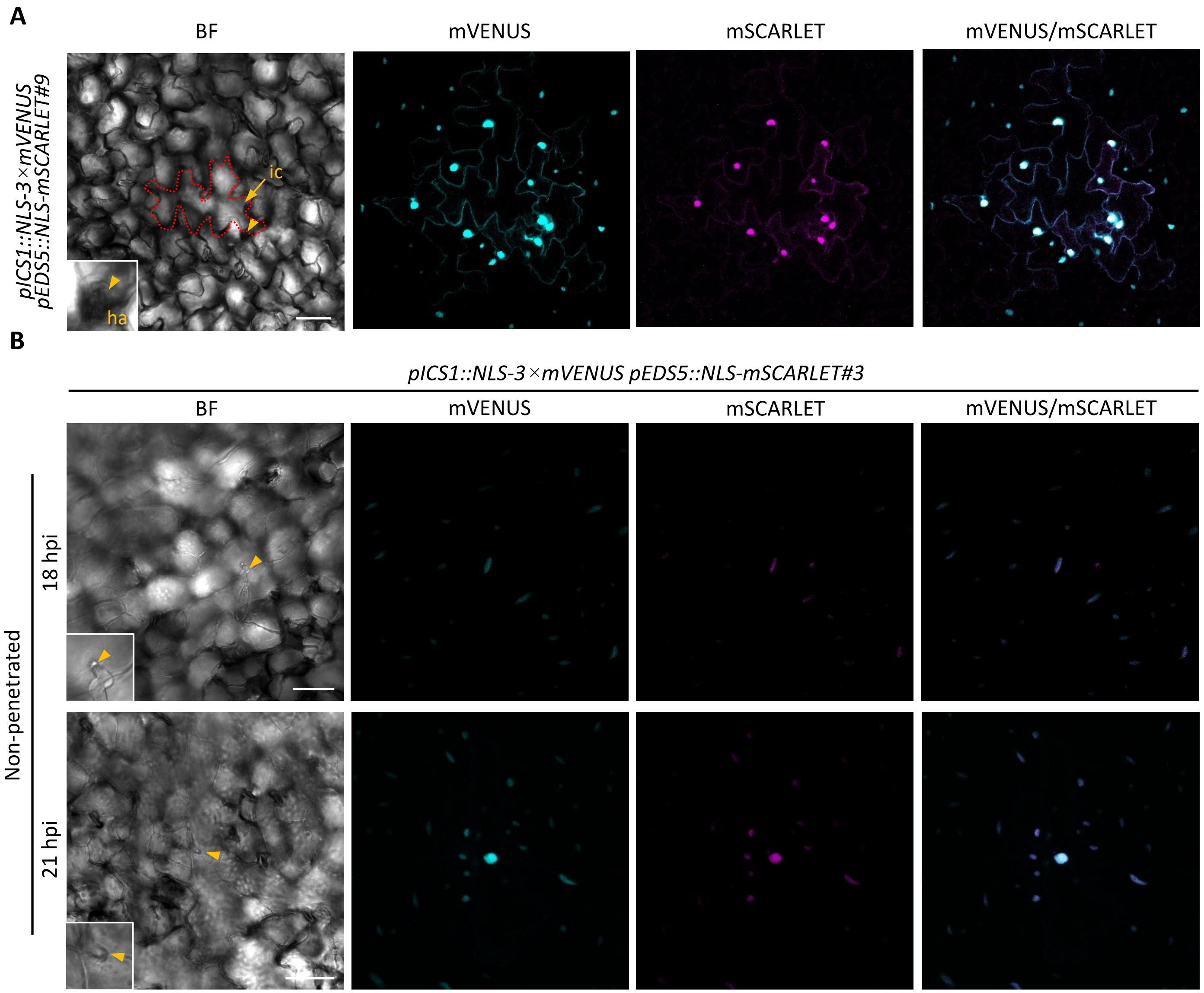

Supplementary Fig. 3 Temporal expression patterns of *ICS1* and *EDS5* promoters in *Arabidopsis* leaves during *B. hordei* infection.

A, Co-expression patterns of the double reporter constructs *pICS1::NLS-3×mVENUS* and *pEDS5::NLS-mSCARLET*#9 in leaf tissues surrounding the *B. hordei*-penetrated site at 24 hpi. Experiments were repeated three times, with at least ten single-infection sites examined. Images are shown as maximal intensity projections of Z-stacks. mVENUS signals are depicted in cyan and mSCARLET in magenta. Infected cells are outlined with red dash lines, and infection sites indicated by arrowheads are magnified enlarged for view. Scale bars, 50 µm. **B**, Confocal micrographs showing representative expression patterns of *pICS1::NLS-3×mVENUS* and *pEDS5::NLS-mSCARLET*#3 in Arabidopsis leaf tissues surrounding *B. hordei* penetration attempts during a time course at 18 and 21 hpi. Fluorescent signals are shown as maximal intensity projections of Z-stacks: mVENUS (*NLS-3×mVENUS*) in cyan and mSCARLET (*NLS-mSCARLET*) in magenta. At least 10 single-infection attempts were examined at each time point. Infection attempts are marked with arrowheads and shown at higher magnification. Scale bars, 50 µm.

Supplementary Fig. 4

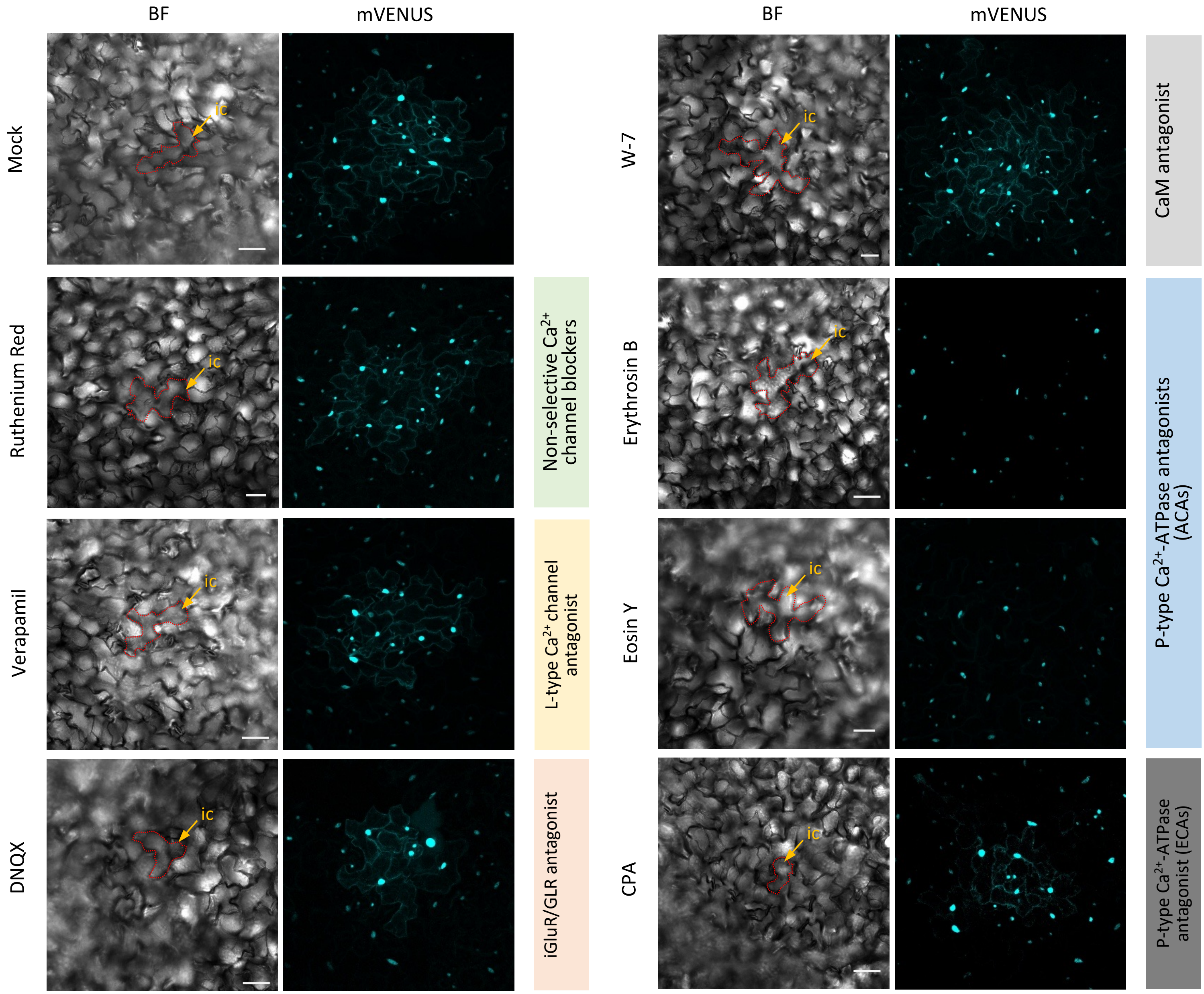

Supplementary Fig. 4 Impact of Ca^{2+} blockers on the *ICS1* expression pattern during *B. hordei* infection.

Representative confocal images showing the expression pattern of the *ICS1* reporter at 24 hpi with *B. hordei* with various Ca^{2+} signaling antagonists treatments. Images are displayed as maximal intensity projections of Z-stacks. Plants infiltrated with water served as the mock control. Experiments were repeated three times, with at least ten single-infection sites examined per treatment in each replicate. mVENUS signals are shown in cyan. Infected cells outlined with red dashed lines. ic, infected cell. Scale bars, 50 μm.

Supplementary Fig. 5

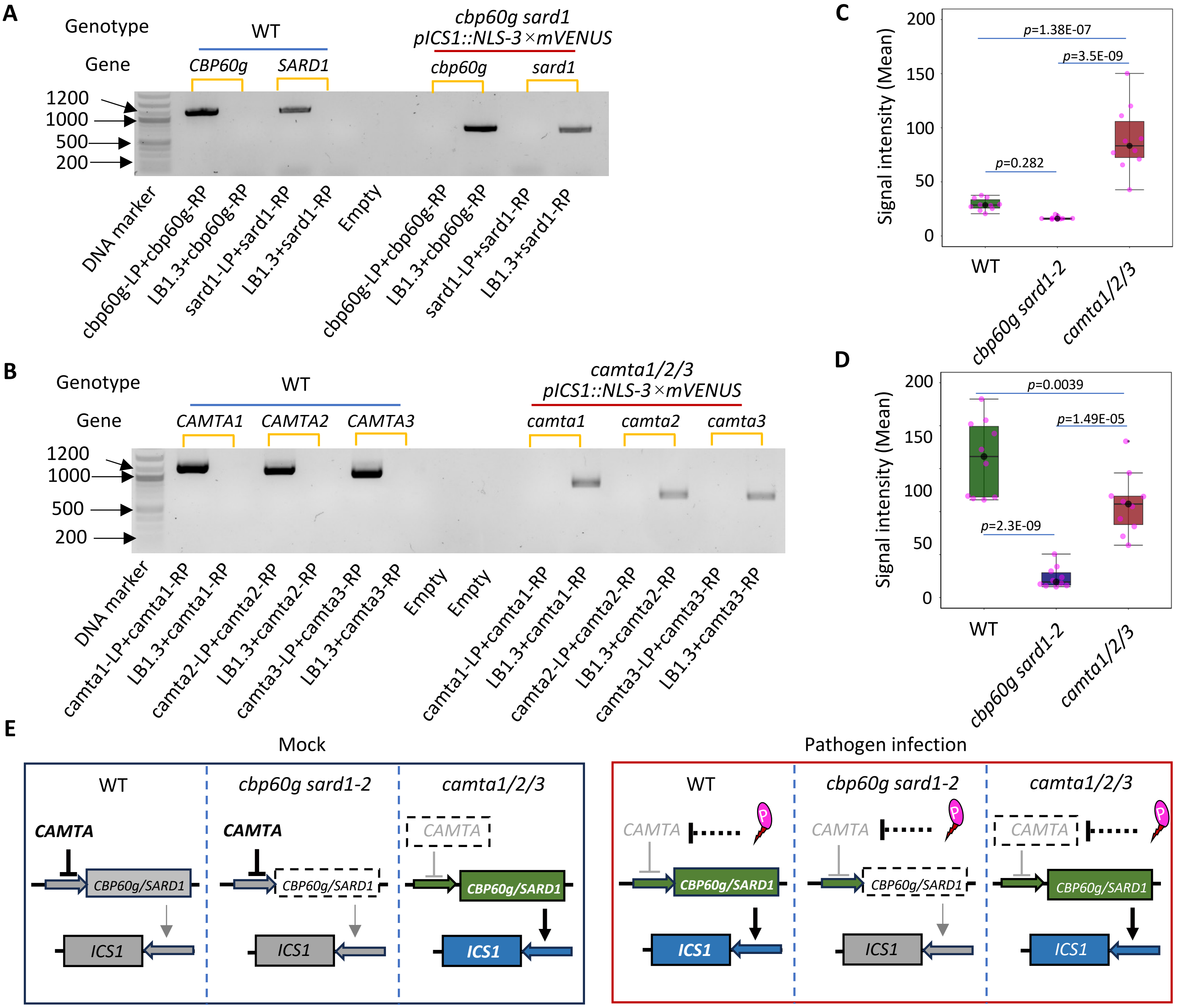

Supplementary Fig. 5 Genotypic verification and quantitative analysis of *pICS1::NLS-3×mVENUS* reporter activity in *cbp60g sard1-2* and *camta1/2/3* mutant backgrounds.

A-B, Gel electrophoresis images of PCR-based genotyping of the *cbp60g* and *sard1* loci in the *cbp60g sard1-2 pICS1::NLS-3×mVENUS* plants (A), and of the *camta1*, *camta2*, and *camta3* loci in the *camta1/2/3* pICS1::*NLS-3×mVENUS* plants (B). WT plants were used as controls. Uncropped gel images are provided as a Source Data file. **C**, Quantitative analysis of nuclear mVENUS fluorescence intensities of the *ICS1* reporter in the WT, *cbp60g sard1-2*, and *camta1/2/3* backgrounds under non-infection conditions. Fluorescence intensity was calculated as average mean nuclear signal per image area. **D**, Quantitative analysis of nuclear mVENUS fluorescence intensities of the *ICS1* reporter in the WT, *cbp60g sard1-2*, and *camta1/2/3* backgrounds at 24 hpi following *B. hordei* penetration. Fluorescence intensity was calculated as the average mean intensities of nuclear mVENUS in the *B. hordei*-penetrated cells and their first layer neighboring cells. **C-D**, All boxplots show the median (center line, n=10), second to third (25% to 75%) quartiles (box), minimum and maximum values (whiskers) of measurement. The statistically significant differences were determined by one-way ANOVA, followed by the post hoc Tukey multiple comparison tests ($p < 0.05$). All p values are indicated. Source data are provided as a Source Data file. **E,** Diagrams illustrating the transcriptional activity of *ICS1* in wild type (WT), *cbp60g sard1-2*, and *camta1/2/3* mutant backgrounds, under both mock and pathogen-infected conditions.

Supplementary Fig. 6

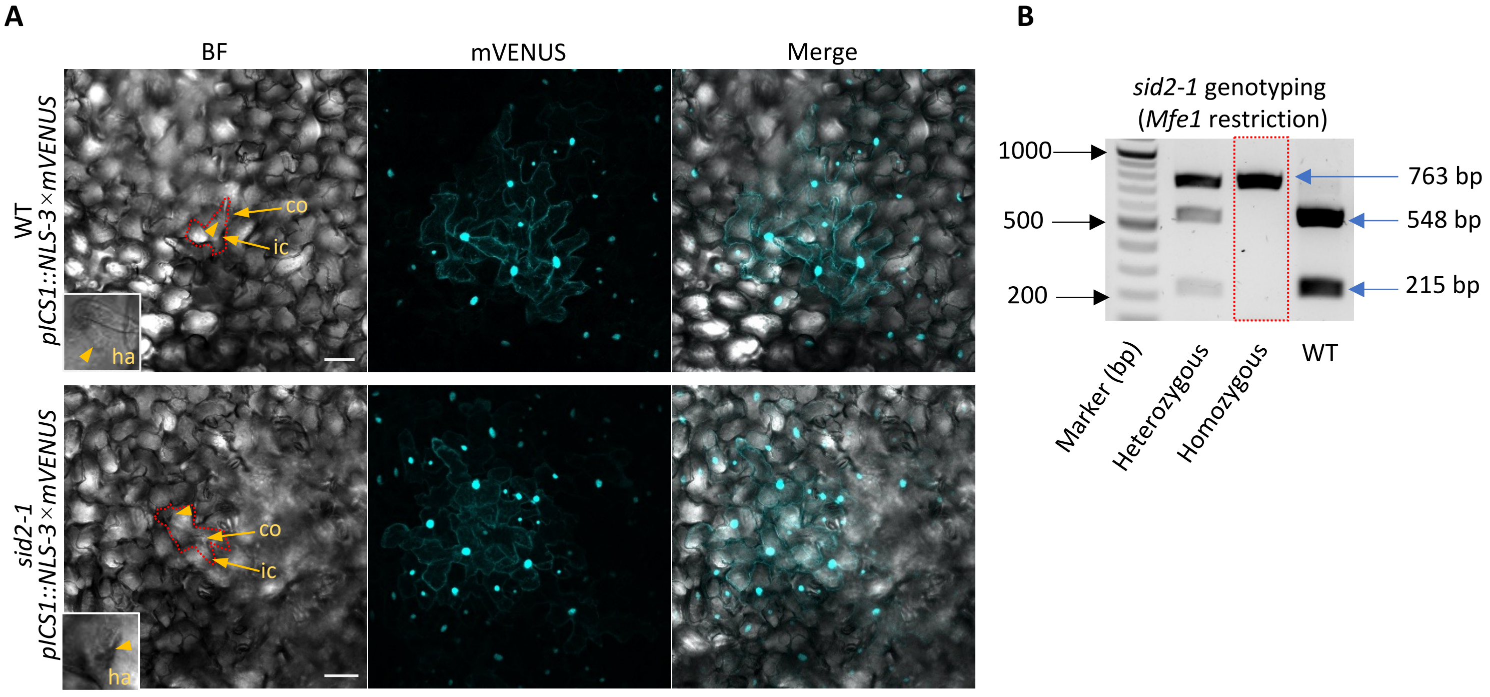

Supplementary Fig. 6 ICS1 promoter activity in Arabidopsis *sid2-1* leaves challenged with *B. hordei*.

A, Representative confocal images showing the expression patterns of *ICS1* (mVENUS, cyan) reporter in Arabidopsis plants with and without the *sid2-1* mutation at 24 hpi with *B. hordei*. Images are maximal intensity projections of Z-stacks. At least ten independent single-infection sites were analyzed. Infected cells outlined with red dashed lines. ic, infected cell; ha, haustorium. Scale bars, 50 µm. **B**, Gel electrophoresis image of PCR-based genotyping of the *ics1* locus in the *sid2-1 pICS1::NLS-3×mVENUS* plants. WT plants were used as controls. Uncropped gel image is provided as a Source Data file.

Supplementary Fig. 7

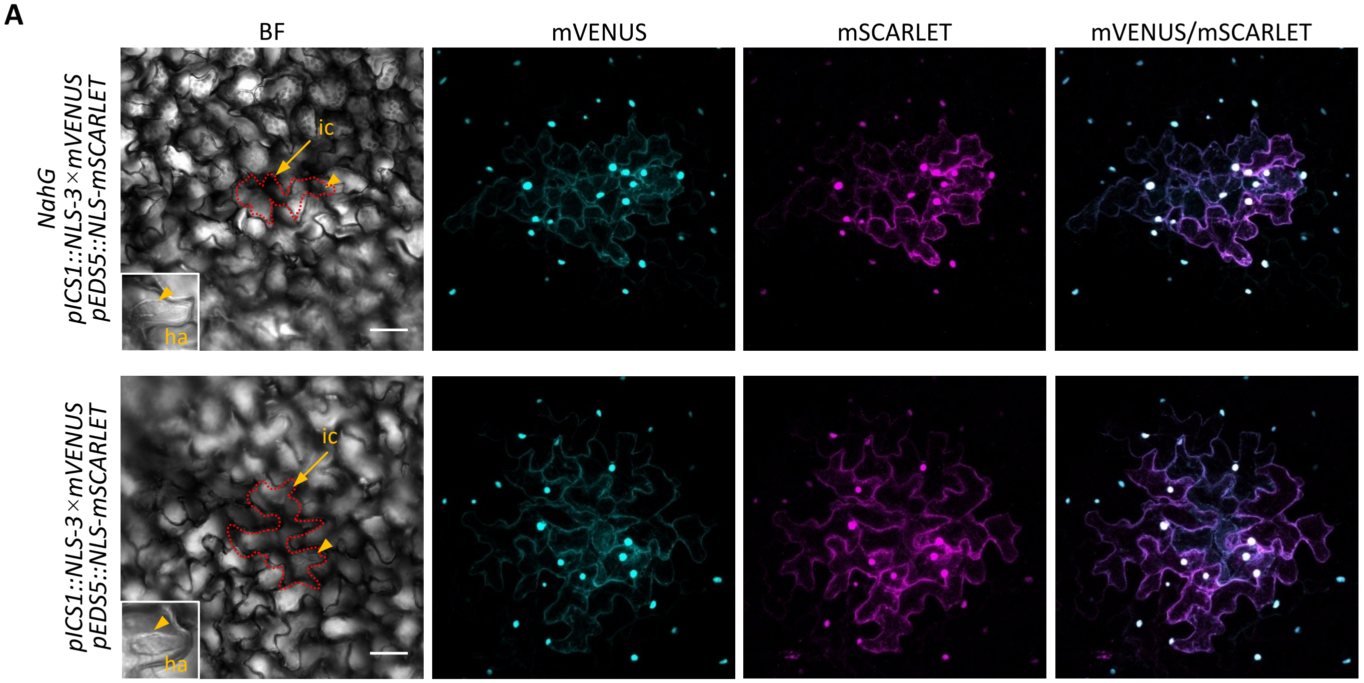

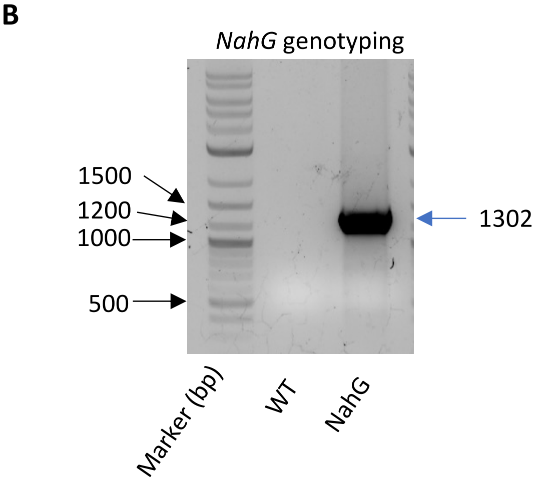

Supplementary Fig. 7 Impact of *NahG* on *ICS1* and *EDS5* promoter activity in *Arabidopsis* leaves challenged with *B. hordei*.

A, Representative confocal images showing the expression patterns of *ICS1* (mVENUS, cyan) and *EDS5* (mSCARLET, magenta) reporters in transgenic Arabidopsis plants with and without the *NahG* background at 24 hpi with *B. hordei*. Images are maximal intensity projections of Z-stacks. At least ten independent single-infection sites were analyzed. Infected cells outlined with red dashed lines. ic, infected cell; ha, haustorium. Scale bars, 50 µm. **B**, Gel electrophoresis image of PCR-based genotyping of the presence of *NahG* in the *NahG pICS1::NLS-3×mVENUS pEDS5::NLS-mSCARLET* plants. WT plants were used as controls. Uncropped gel image is provided as a Source Data file.

Supplementary Fig. 8

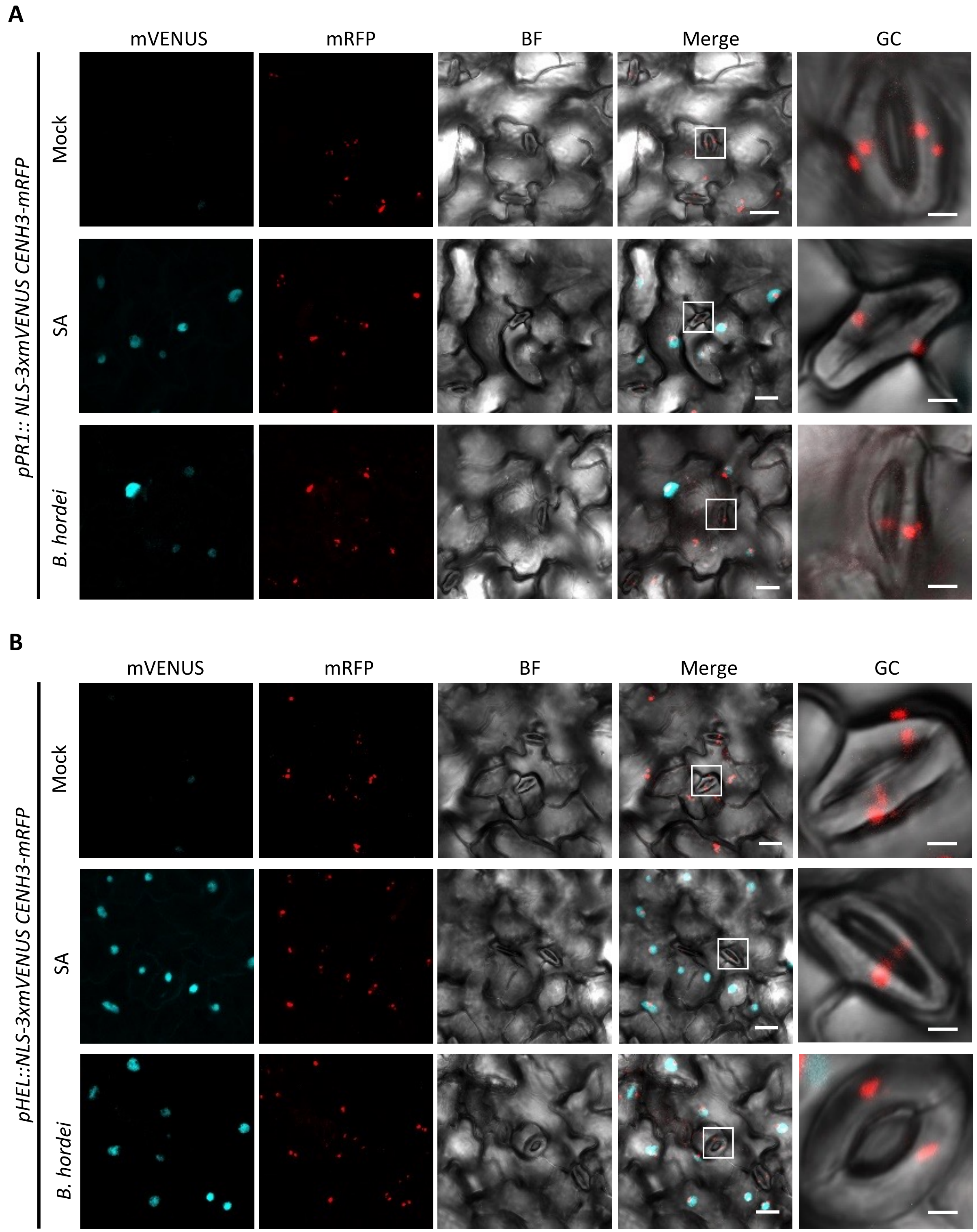

Supplementary Fig. 8 Expression dynamics of immune marker genes in Arabidopsis guard cells under SA treatment and *B. hordei* infection.

A-B, Representative confocal images showing expression of immune marker genes in transgenic plants: *pPR1::NLS-3×mVENUS CENH3-mRFP* (A) and *pHEL::NLS-3×mVENUS CENH3-mRFP* (B) treated with salicylic acid (SA) spray or *B. hordei* inoculation. Images represent maximal intensity projections of Z-stacks. Each treatment was performed in triplicate, with at least 10 single-infection sites analyzed per treatment per replicate. mVENUS signals are shown in cyan, and mRFP signals (CENH3 nuclear marker) are shown in red. Scale bars, 20 μm. Enlarged view of guard cells adjacent to infected pavement cells are shown in the right panels. Scale bars, 2 μm.

Supplementary Fig. 9

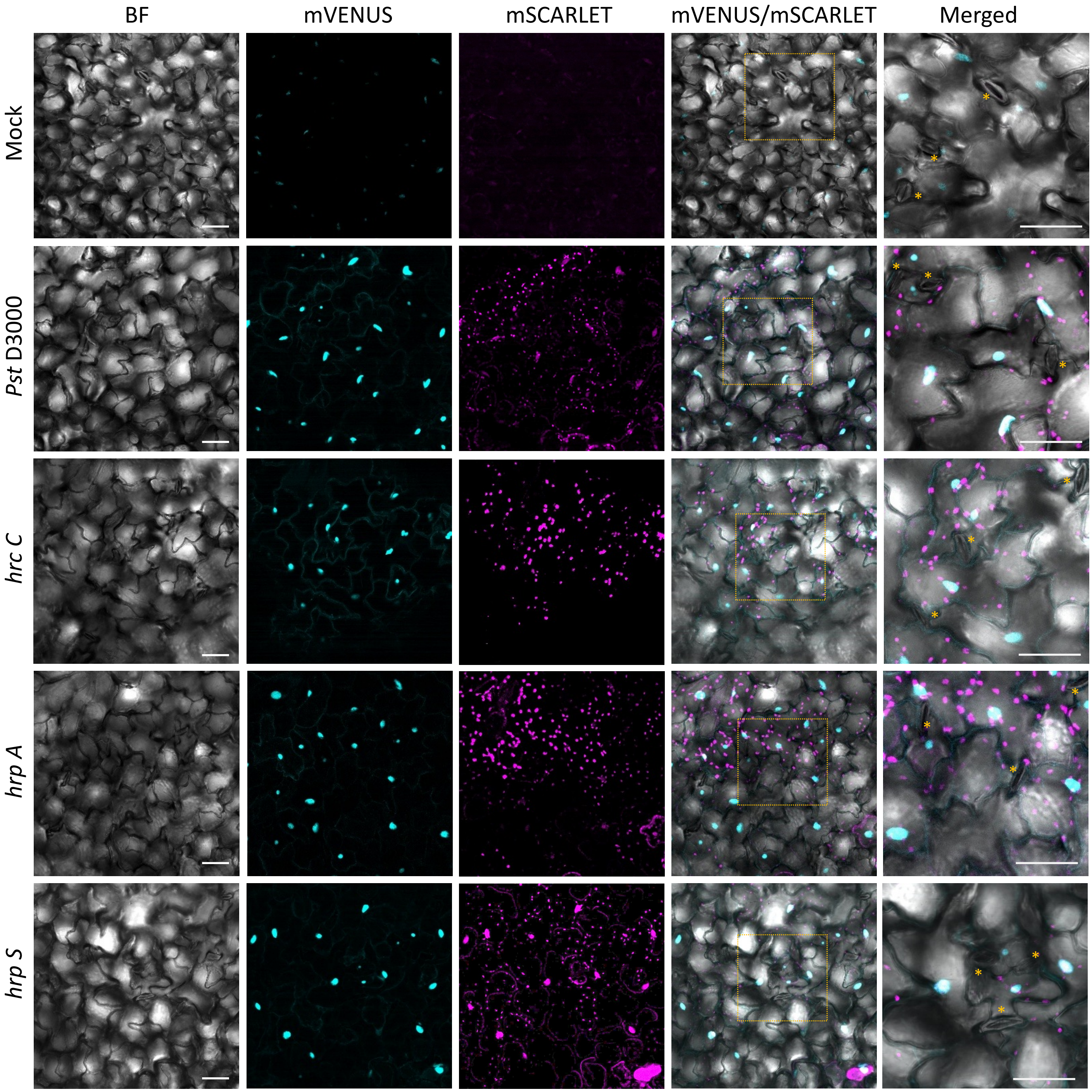

Supplementary Fig. 9 Co-expression patterns of *ICS1* in leaf tissues upon infection by various *Pseudomonas syringae* pv *tomato* strains.

Representative image showing expression patterns of *pICS1::NLS-3×mVENUS* and *pICS1:ICS1-mSCARLET* in pavement and guard cells following infiltration with *P. syringae* virulent WT and *hrp*-deficient (*hrc C, hrp A,* and *hrp S*) strains. Leaf tissues infiltrated with water were used as mock controls. The enlarged inset provides a close-up view of the infected guard cells. Guard cells are indicated by asterisks. Observations were made at a minimum of three biological repeats. Fluorescent images are presented as maximal intensity projections of Z-stacks. mVENUS signals appear in in cyan, and mSCARLET signals in magenta. Scale bars, 50 μm.

Supplementary Fig. 10

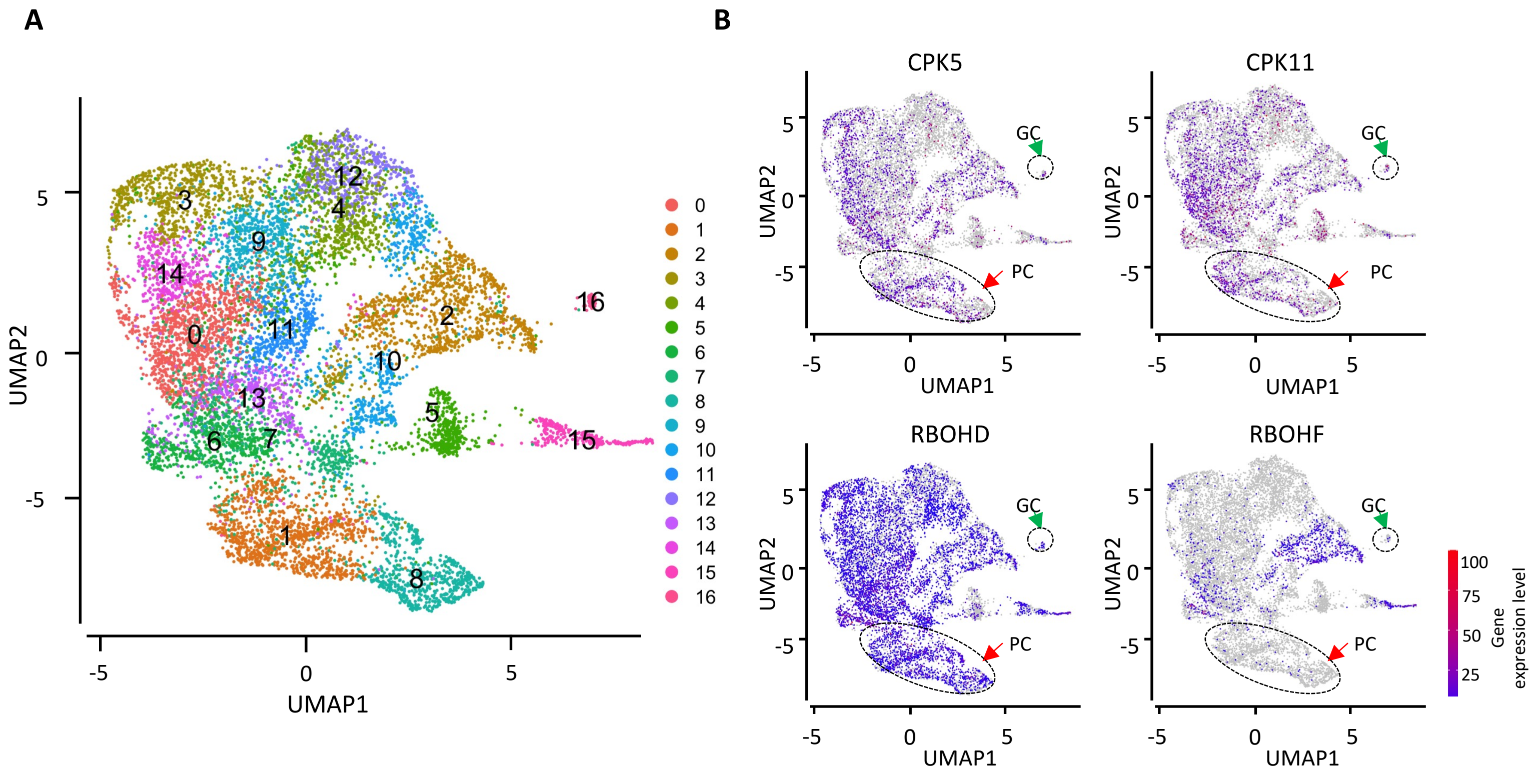

Supplementary Fig. 10 Identification of cell clusters and expression of genes involved in ROS production in response to *P. syringae*.

A, UMAP projection of the 16 cell clusters obtained using principle-component analysis in response to *P. syringae* (Data collected from published datasets, NCBI Bio-project: PRJNA995336). Clusters 1 and 8 are identified as pavement cells (epidermis) and cluster 16 is identified as GCs. **B,** Cell-specific expression profiles of genes involved in ROS production in response to *P. syringae*.

Supplementary Fig. 11

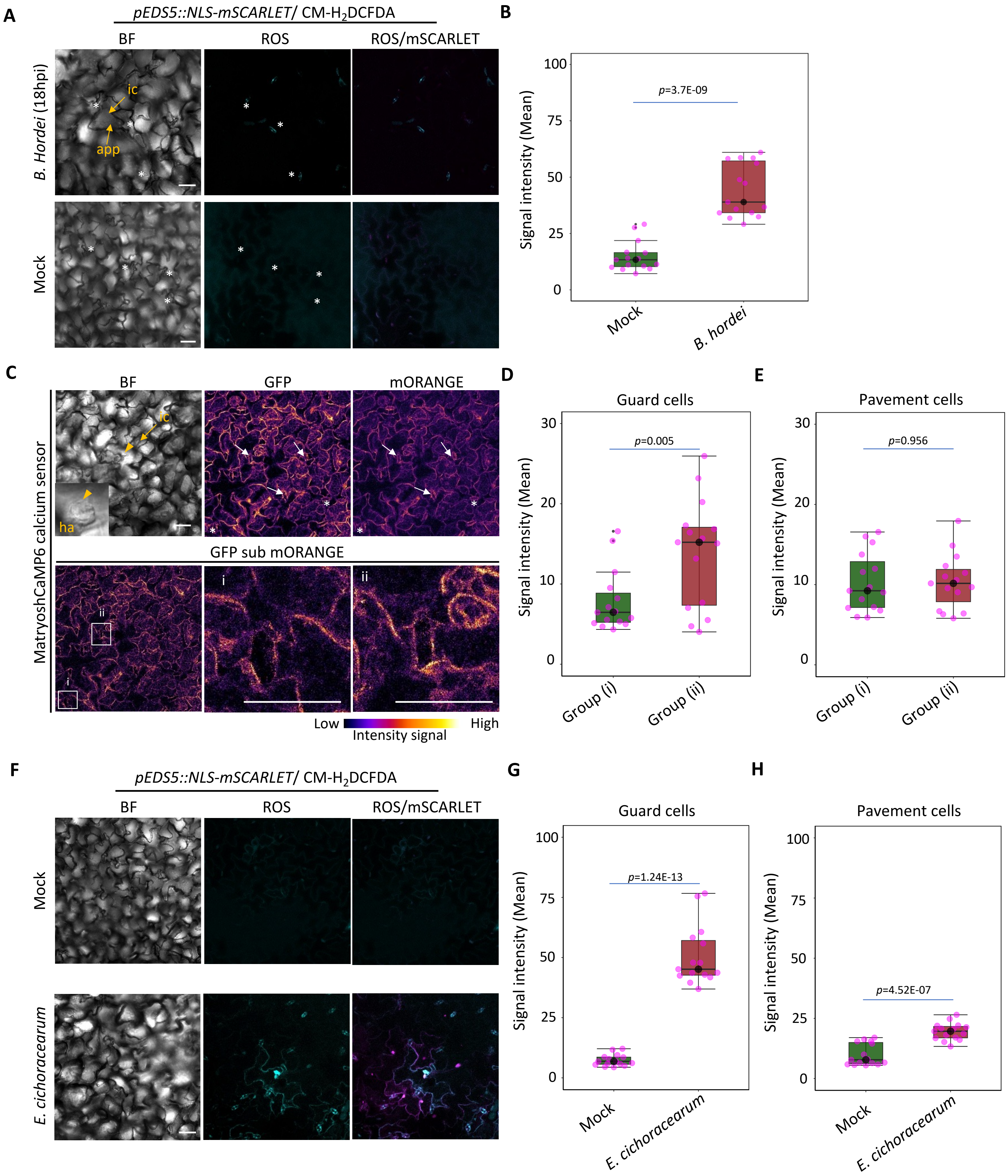

Supplementary Fig. 11 Ca^{2+} elevation and ROS accumulation in guard cells during powdery mildew infection.

A, Representative images showing accumulation of reactive oxygen species (ROS) detected by CM-H_2DCFDA in *B. hordei* infected leaf tissues of *pEDS5:NLS-mSCARLET* plants at 18 hpi. CM-H_2DCFDA staining and mSCARLET fluorescence are shown as maximal intensity projections of Z-stacks. A minimum of ten single-infection sites were examined. White asterisks mark guard cells. ROS signals are displayed in cyan, and mSCARLET signals in magenta. Scale bars, 50 µm. **B**, Boxplot showing quantitative analysis of mean ROS signal intensities in two groups of guard cells (mock and *B. hordei*) as defined in (A). **C**, Cytosolic Ca^{2+} dynamics detected by the calcium sensor MatryoshCaMP6 in *E. cichoracearum* infected leaf tissue at 22 hpi. Fluorescent signals from GFP and mORANGE channels are displayed as maximum intensity projections of Z-stacks. At least ten single-infection sites were examined. The infected cell is outlined with red dash line. White asterisks mark non-immediate neighboring guard cells, white arrows point to immediate neighboring guard cells. Signal intensities for GFP and mORANGE are shown as the mpl-inferno LUT (ImageJ). Scale bars, 50 µm. Enlarged panels show a representative examples of a non-immediate neighbor guard cell (i) and an immediate neighbor guard cell (ii). ic, infected cell; ha, haustorium. Scale bars (i-ii), 50 µm. **D**, Boxplot showing the quantitative analysis of mean GFP/mORANGE signal intensities in two groups of guard cells as defined in (D). Group (i) represents non-immediate neighboring guard cells, and Group (ii) represents immediate neighboring guard cells of the infected pavement cell. **E**, Boxplot showing the quantitative analysis of mean GFP/mORANGE signal intensities in two groups of pavement cells as defined in (D). Group (i) represents non-immediate neighboring pavement cells, and Group (ii) represents immediate neighboring pavement cells of the infected pavement cell. **F**, Accumulation of ROS detected by CM-H_2DCFDA in *E. cichoracearum* infected leaf tissues of *pEDS5:NLS-mSCARLET* plants at 26 hpi. Healthy leaves from non-inoculated plants were used as mock control. CM-H_2DCFDA staining and mSCARLET fluorescence are shown as maximal intensity projections of Z-stacks. A minimum of ten single-infection sites were examined. ROS signals are displayed in cyan, and mSCARLET signals in magenta. Scale bars, 50 µm. **G**, Boxplot showing quantitative analysis of mean ROS signal intensities in two groups of guard cells (mock and *E. cichoracearum*) as defined in (D). **H**, Boxplot showing quantitative analysis of mean ROS signal intensities in two groups of pavement cells (mock and *E. cichoracearum*) as defined in (D). **B**, **D**-**E**, and **G**-**H**, All boxplot show the median (center line, n=15), second to third (25% to 75%) quartiles (box), minimum and maximum values (whiskers) of measurement. Statistical significance was assessed using two-tailed unpaired Student's *t*-test; All *p* values are indicated. Source data are provided as a Source Data file.

Supplementary Fig. 12

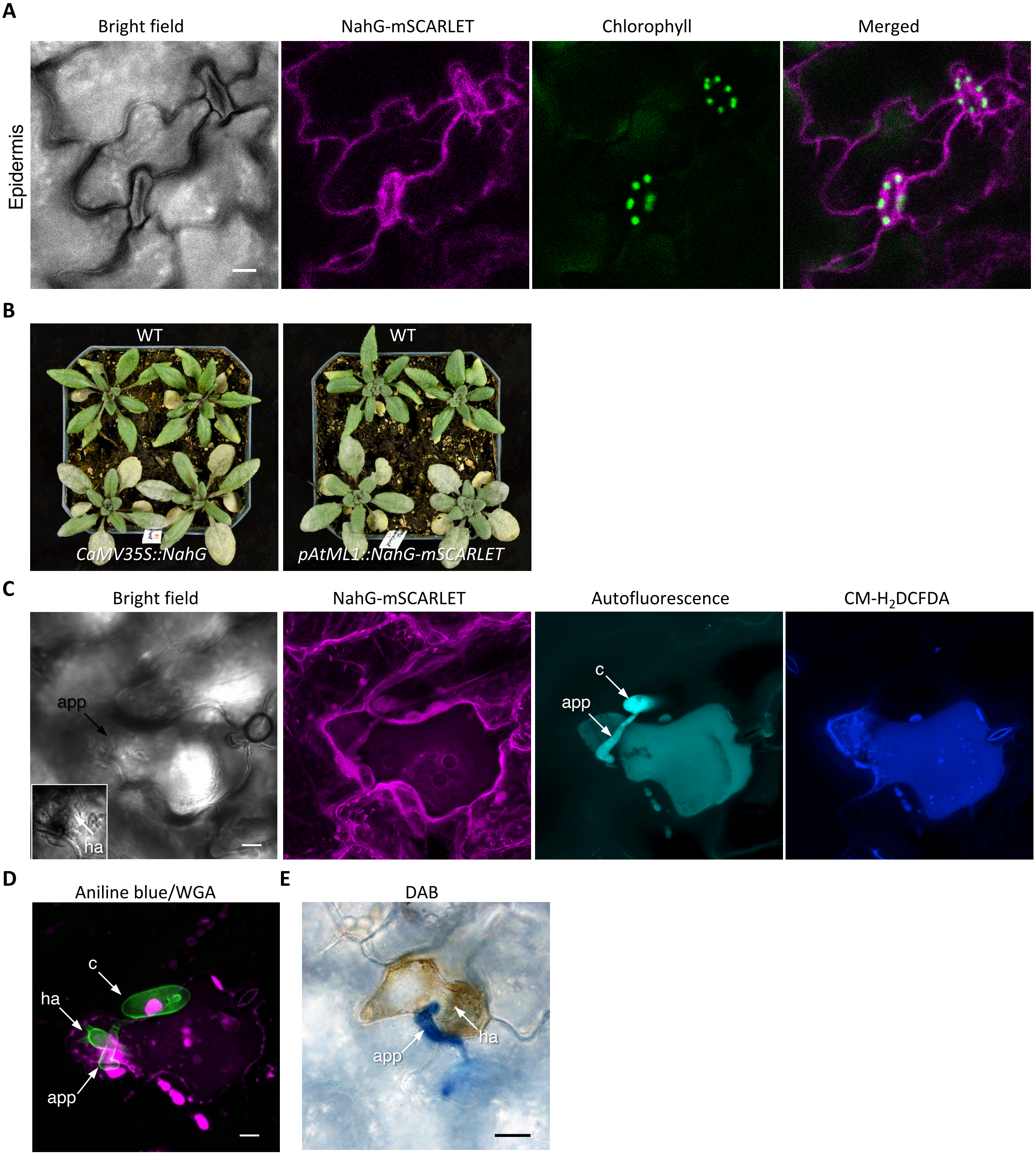

Supplementary Fig. 12. ***B. hordei*** **infection triggers cell death in non-host Arabidopsis independent of salicylic acid signaling.**

A, Targeted expression of *pAtML1::NahG-mSCARLET* in the epidermis of Arabidopsis leaves. Leaf tissues from two-week-old plants was examined by confocal microscopy. Observations were made at a minimum of three biological repeats. mSCARLET fluorescence and chlorophyll autofluorescence were collected by focusing on the epidermal layer. Scale bar, 10 µM. **B**, Symptom development on leaves of wild type (WT) and transgenic lines expressing *CaMV35S::NahG* or *pAtML1::NahG-mSCARLET* at 8 dpi with *E. cichoracearum* conidiospores. Experiments were repeated three times. **C-D,** Immune responses in Arabidopsis plants expressing *pAtML1::NahG-mSCARLET* associated with *B. hordei*-penetrated epidermal cells undergoing hypersensitive cell death. Leaf samples collected at 26 hpi were examined by confocal microscopy. Images show *NahG-mSCARLET* expression*,* ROS accumulation detected with CM-H_2DCFDA, and cell autofluorescence (C). Leaf tissues with the same infection site was fixed and stained with with aniline blue for callose detection and WGA staining for fungal structures (D). A minimum of ten single-infection sites were examined. Scale bar, 10 µM. **E,** ROS accumulation in *B. hordei*-penetrated epidermal cells. Leaf samples at 26 hpi were detached and stained with 3,3′-diaminobenzidine (DAB) for H_2O_2 detection. A minimum of ten single-infection sites were examined. Scale bar, 10 µM. c, conidium; app, appressorium; ha, haustorium.

Supplementary Fig. 13

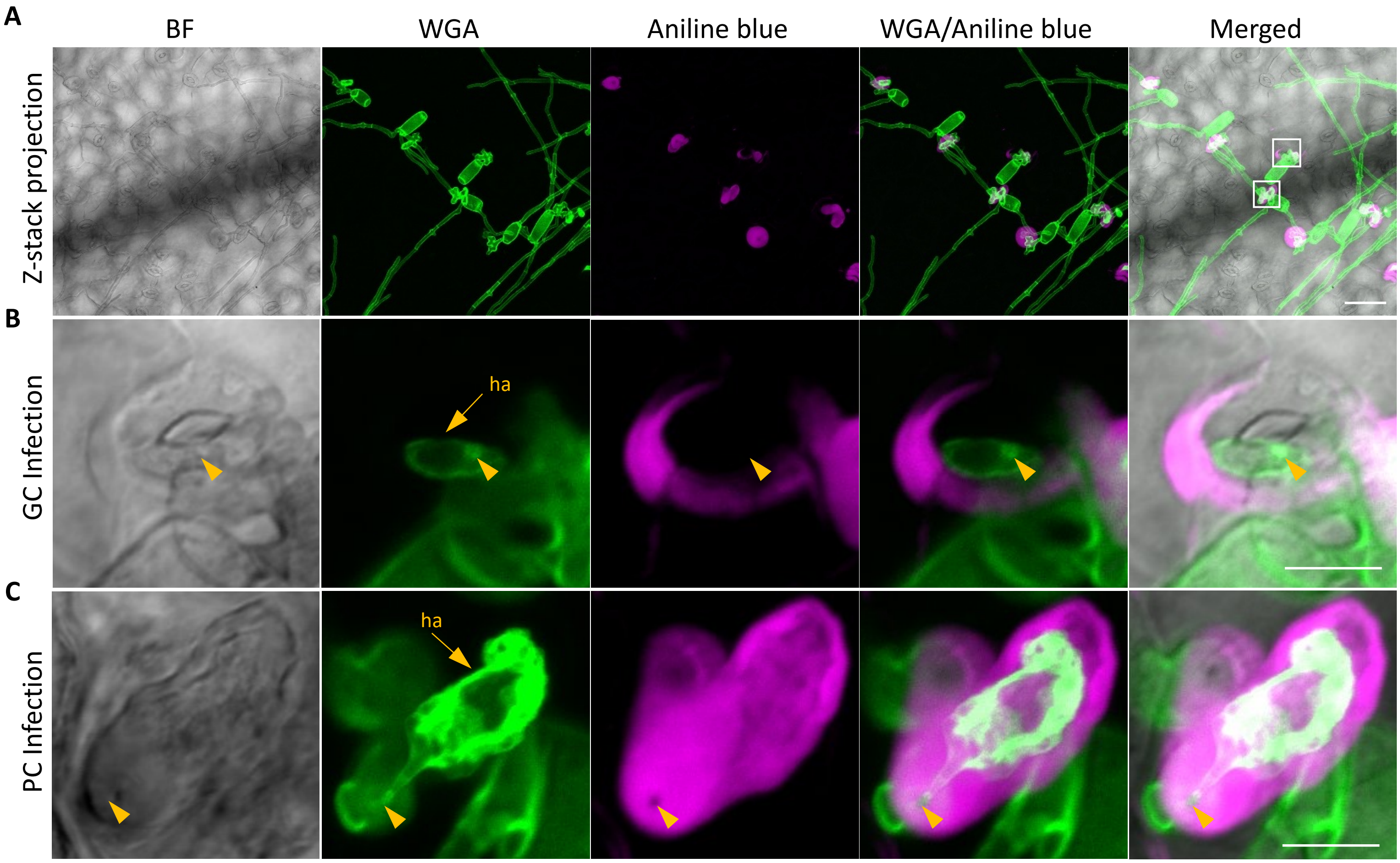

Supplementary Fig. 13 Guard cells are incompatible with host-adapted powdery mildew *Erysiphe cruciferarum*.

(**A**-**C**) Confocal micrographs showing *Erysiphe cruciferarum* (*E. cruciferarum*) conidiospores and penetration structures in epidermal cells of Arabidopsis leaves at 48 hpi. At least ten independent single-infection sites per cell type were examined. Fungal structures were visualized using wheat germ agglutinin conjugated to Alexa Fluor 488 (WGA, green), and callose deposition at infection sites was detected by aniline blue staining (magenta). Images are shown as Z-stack maximum intensity projections acquired by confocal microscopy. Scale bar, 50 µm. **B**, Enlarged single-plane image showing the *E. cruciferarum* penetration in a guard cell from (**A**). Scale bar, 10 µm. **C**, Enlarged single-plane image showing the *E. cruciferarum* penetration in pavement cells from (**A**). Scale bar, 10 µm. Penetration sites are indicated by arrow heads. GC, guard cell; PC, pavement cell. Haustoria (ha) are indicated by arrows.

Supplementary Fig. 14

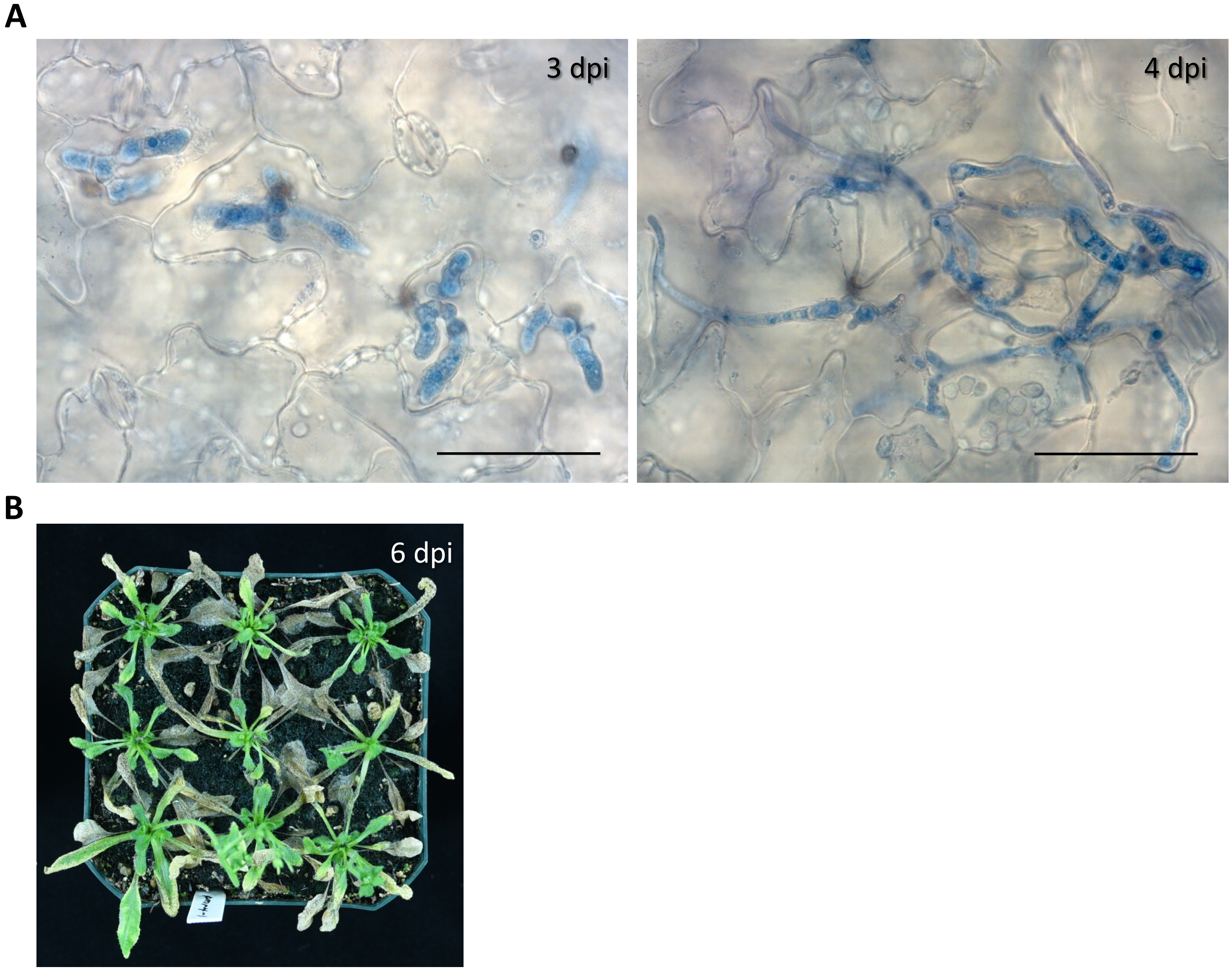

Supplementary Fig. 14 ***Colletotrichum higginsianum*** **is a host-adapted pathogen of** ***Arabidopsis*****.**

A, Representative microscopic images of *C. higginsianum*-infected Arabidopsis leaf tissues at 3 and 4 days post inoculation (dpi), stained with trypan blue to visualize fungal structures. Scale bar, 50 µm. **B,** Disease symptoms in Col-0 Arabidopsis leaves at 6 dpi showing advanced stages of *C. higginsianum* infection and lesion development. Experiments were performed with three biological replicates.

Supplementary Table 1. Primers used for this study

Primer ID	Sequence	Experiment
Pac1-mScarlet-F	GGTTAATTAAAATGGTGAGCAAGGGCGAG	mSCARLET gene cloning
Spe1-mScarlet-R	CCACTAGTGATCTAGTAACATAGATGACACCGCG	
Asc1-pICS1-F	TGGCGCGCCAAATTATCCACGCTTTGTCAC	pICS1::ICS1 cloning
Pac1-gICS1-R	GGTTAATTAAATTAATCGCCTGTAGAGATG	
Asc1-pEDS5-F	TGGCGCGCCGGGCAGAAGGAGAATGTAAAG	EDS5 promoter cloning
Pac1-NLS-pEDS5-R	GGTTAATTAAGTCCAACTTGACCCTCTTGGCAGCAGGCATTTTGA GAAAAATCGGTGAATC	
sid2-LP	GTTCTCTATCGTACGAGAG	*sid2-1* genotyping
sid2-RP	TAGATCAATGCCCCAAGACC	
NahG-LP	ATGAAAAACAATAAACTTGGCTTGCG	*NahG* genotyping
NahG-RP	CCCTTGACGTAGCACACC	
cbp60g-LP	TCAATGAAGATTCGGAACAGC	*cbp60g* genotyping
cbp60g-RP	ACTTCCGACTCCTAGTCCAGC	
sard1-LP	CCAAATGTGGTCAGGTCAAAG	*sard1* genotyping
sard1-RP	CGATGTCTAGTGTGCGAATTG	
camta1-LP	AGGGTGGGGAGATAATGTCAG	*camta1* genotyping
camta1-RP	TACGTGTCGGAGGTTTATTGG	
camta2-LP	GGAACCTCCACTTCTCCAAAC	*camta2* genotyping
camta2-RP	CCCTGTTAACGTCAGAGCATC	
camta3-LP	TGAAAACCTGATGAATCCGAG	*camta3* genotyping
camta3-RP	TGTTTGGGCAAACAGAAGTTC	
LBb1.3	ATTTTGCCGATTTCGGAAC	T-DNA specific primer
Asc1-NahG-FOR	TTGGCGCGCCATGAAAAACAATAAACTTGGCTTGCG	NaHG amplification
Pac1-NahG-REV	GGTTAATTAACCCTTGACGTAGCACACC	
Pme1-AtML1-FOR	CCGTTTAAACCATTTACACATCCTGTCG	AtML1 promoter amplification
Asc1-AtML1-REV	TTGGCGCGCCGTGGATTCAGGGAGTTTC	
