# Supplementary material for: Cell-type-specific immune programs orchestrate spatial defense in the Arabidopsis leaf epidermis
Source: Nat Commun. 2026 Mar 21;17:4296. doi: 10.1038/s41467-026-70843-z (PMC13172538; doi:10.1038/s41467-026-70843-z)
Supplement: Supplementary file 2 — Description of Additional Supplementary Files [file 41467_2026_70843_MOESM2_ESM.pdf]

Description of Additional Supplementary Files

File Name: Supplementary Movie 1

Description: Spatial distribution of mVENUS signals in ICS1 reporter leaf epidermis surrounding a *B. hordei* penetration site.

File Name: Supplementary Movie 2

Description: Callose deposition associated with a *B. hordei*-penetrated pavement cell.

File Name: Supplementary Movie 3

Description: Callose deposition associated with a *B. hordei*-penetrated guard cell.

File Name: Supplementary Movie 4

Description: *E. cichoracearum*-penetrated pavement cell.

File Name: Supplementary Movie 5

Description: *E. cichoracearum*-penetrated guard cell.

File Name: Supplementary Movie 6

Description: *E. cruciferarum* infection in Arabidopsis pavement cell and guard cell.

File Name: Supplementary Movie 7

Description: *C. higginsianum* infection in Arabidopsis pavement cell and guard cell.
