# Supplementary material for: Cell-type-specific immune programs orchestrate spatial defense in the Arabidopsis leaf epidermis
Source: Nat Commun. 2026 Mar 21;17:4296. doi: 10.1038/s41467-026-70843-z (PMC13172538; doi:10.1038/s41467-026-70843-z)
Supplement: Supplementary file 10 — Reporting Summary [file 41467_2026_70843_MOESM10_ESM.pdf]

nature portfolio

Corresponding author(s): Yangdou Wei

Last updated by author(s): Feb 17, 2026

# Reporting Summary

Nature Portfolio wishes to improve the reproducibility of the work that we publish. This form provides structure for consistency and transparency in reporting. For further information on Nature Portfolio policies, see our Editorial Policies and the Editorial Policy Checklist.

## Statistics

For all statistical analyses, confirm that the following items are present in the figure legend, table legend, main text, or Methods section.

| n/a | Confirmed | |
|---|---|---|
| ☐ | ☒ | The exact sample size (*n*) for each experimental group/condition, given as a discrete number and unit of measurement |
| ☐ | ☒ | A statement on whether measurements were taken from distinct samples or whether the same sample was measured repeatedly |
| ☐ | ☒ | The statistical test(s) used AND whether they are one- or two-sided<br>*Only common tests should be described solely by name; describe more complex techniques in the Methods section.* |
| ☒ | ☐ | A description of all covariates tested |
| ☒ | ☐ | A description of any assumptions or corrections, such as tests of normality and adjustment for multiple comparisons |
| ☐ | ☒ | A full description of the statistical parameters including central tendency (e.g. means) or other basic estimates (e.g. regression coefficient) AND variation (e.g. standard deviation) or associated estimates of uncertainty (e.g. confidence intervals) |
| ☐ | ☒ | For null hypothesis testing, the test statistic (e.g. *F*, *t*, *r*) with confidence intervals, effect sizes, degrees of freedom and *P* value noted<br>*Give P values as exact values whenever suitable.* |
| ☒ | ☐ | For Bayesian analysis, information on the choice of priors and Markov chain Monte Carlo settings |
| ☒ | ☐ | For hierarchical and complex designs, identification of the appropriate level for tests and full reporting of outcomes |
| ☒ | ☐ | Estimates of effect sizes (e.g. Cohen's *d*, Pearson's *r*), indicating how they were calculated |

*Our web collection on statistics for biologists contains articles on many of the points above.*

## Software and code

Policy information about availability of computer code

| | |
|---|---|
| Data collection | Confocal images were analyzed using the imaging software: LSM Image Browser (Zeiss) and Fiji ImageJ (2.14.0) software. |
| Data analysis | All statistical analyses were performed using R software (version 4.1.2; http://r-project.org). Comparisons between two groups were conducted using two-tailed Student’s t-tests. Box plots were generated in RStudio using appropriate data visualization packages. |

For manuscripts utilizing custom algorithms or software that are central to the research but not yet described in published literature, software must be made available to editors and reviewers. We strongly encourage code deposition in a community repository (e.g. GitHub). See the Nature Portfolio guidelines for submitting code & software for further information.

## Data

Policy information about availability of data

All manuscripts must include a data availability statement. This statement should provide the following information, where applicable:

- Accession codes, unique identifiers, or web links for publicly available datasets
- A description of any restrictions on data availability
- For clinical datasets or third party data, please ensure that the statement adheres to our policy

All constructs and transgenic lines generated in this study are available from the corresponding author upon request. Source data are provided with this paper.

## Research involving human participants, their data, or biological material

Policy information about studies with human participants or human data. See also policy information about sex, gender (identity/presentation), and sexual orientation and race, ethnicity and racism.

| | |
|---|---|
| Reporting on sex and gender | No reporting on sex and gender was referenced in this manuscript |
| Reporting on race, ethnicity, or other socially relevant groupings | No reporting on race, ethnicity, or other socially relevant groupings. |
| Population characteristics | NA |
| Recruitment | NA |
| Ethics oversight | NA |

Note that full information on the approval of the study protocol must also be provided in the manuscript.

# Field-specific reporting

Please select the one below that is the best fit for your research. If you are not sure, read the appropriate sections before making your selection.

- [x] Life sciences
- [ ] Behavioural & social sciences
- [ ] Ecological, evolutionary & environmental sciences

For a reference copy of the document with all sections, see nature.com/documents/nr-reporting-summary-flat.pdf

# Life sciences study design

All studies must disclose on these points even when the disclosure is negative.

| | |
|---|---|
| Sample size | Sample sizes were determined based on our previous experience with similar experiments and guidance from published literature. For each chemical treatment or pathogen challenge, three biological replicates were typically included. Specific sample sizes for individual experiments are provided in the corresponding figure legends. |
| Data exclusions | No data were excluded from the analysis. |
| Replication | All attempts at replication were successful. |
| Randomization | All samples were randomly assigned to experimental groups. Biological replicates were grown and collected independently at different times; however, all samples were processed simultaneously to minimize technical variation. |
| Blinding | To ensure the independence of biological replicates, plant sample collection, experimental procedures, and data analysis were carried out by researchers not directly involved in this project. |

# Reporting for specific materials, systems and methods

We require information from authors about some types of materials, experimental systems and methods used in many studies. Here, indicate whether each material, system or method listed is relevant to your study. If you are not sure if a list item applies to your research, read the appropriate section before selecting a response.

### Materials & experimental systems

| n/a | Involved in the study | |
|---|---|---|
| x | | Antibodies |
| x | | Eukaryotic cell lines |
| x | | Palaeontology and archaeology |
| x | | Animals and other organisms |
| x | | Clinical data |
| x | | Dual use research of concern |
| | x | Plants |

### Methods

| n/a | Involved in the study | |
|---|---|---|
| x | | ChIP-seq |
| x | | Flow cytometry |
| x | | MRI-based neuroimaging |

## Dual use research of concern

Policy information about dual use research of concern

### Hazards

Could the accidental, deliberate or reckless misuse of agents or technologies generated in the work, or the application of information presented in the manuscript, pose a threat to:

| No | Yes | |
|---|---|---|
| ☒ | ☐ | Public health |
| ☒ | ☐ | National security |
| ☒ | ☐ | Crops and/or livestock |
| ☒ | ☐ | Ecosystems |
| ☒ | ☐ | Any other significant area |

### Experiments of concern

Does the work involve any of these experiments of concern:

| No | Yes | |
|---|---|---|
| ☒ | ☐ | Demonstrate how to render a vaccine ineffective |
| ☒ | ☐ | Confer resistance to therapeutically useful antibiotics or antiviral agents |
| ☒ | ☐ | Enhance the virulence of a pathogen or render a nonpathogen virulent |
| ☒ | ☐ | Increase transmissibility of a pathogen |
| ☒ | ☐ | Alter the host range of a pathogen |
| ☒ | ☐ | Enable evasion of diagnostic/detection modalities |
| ☒ | ☐ | Enable the weaponization of a biological agent or toxin |
| ☒ | ☐ | Any other potentially harmful combination of experiments and agents |

## Plants

| | |
|---|---|
| Seed stocks | Arabidopsis Biological Resources Center (cbp60g sard1-2, CS72191; eds5-1, CS3735; camta1 camta2 camta3, CS71606; pICS1::NLS-3XmVENUS, CS2107988; pPR1::NLS-3XmVENUS, CS2107991; pHEL::NLS-3XmVENUS, CS2107982; pLipoP1::NLS-3XmCITRINE, CS73266; pFRK1::NLS-3XmVENUS, CS2110219; N7 nuclear marker, CS84731; PT-CK, CS16265; MatryoshCaMP6s, CS69957) and Nottingham Arabidopsis Stock Centre (CENH3-mRFP, N799456). Transgenic plants pICS1::ICS1-mSCARLET sid2-1,pEDS5::NLS-mSCARLET and pAtML1::NanG-mScarlet were generated in our laboratory and can be obtained by contacting the corresponding author. |
| Novel plant genotypes | pICS1::ICS1-mSCARLET sid2-1 and pEDS5::NLS-mSCARLET plants were generated using Agrobacterium-mediated transformation methods and at least two positive lines were selected for experiments. |
| Authentication | T-DNA insertion lines were genotyped by PCR, and EMS-induced point mutations were confirmed by Sanger sequencing. Transgenic plants generated in this study were selected using Basta resistance, and fluorescent signals were verified by confocal microscopy. |
